# Progression of a large syphilis outbreak in rural North Carolina through space and time: Application of a Bayesian Maximum Entropy graphical user interface

**DOI:** 10.1371/journal.pgph.0001714

**Published:** 2023-05-04

**Authors:** Lani C. Fox, William C. Miller, Dionne Gesink, Irene Doherty, Kristen H. Hampton, Peter A. Leone, Delbert E. Williams, Yasuyuki Akita, Molly Dunn, Marc L. Serre

**Affiliations:** 1 Department of Environmental Sciences and Engineering, Gillings School of Global Public Health, University of North Carolina at Chapel Hill, Chapel Hill, North Carolina, United States of America; 2 Lani Fox Geostatistical Consulting, Claremont, California, United States of America; 3 Department of Epidemiology, Gillings School of Global Public Health, University of North Carolina at Chapel Hill, Chapel Hill, North Carolina, United States of America; 4 School of Medicine, Division of Infectious Diseases University of North Carolina at Chapel Hill, Chapel Hill, North Carolina, United States of America; 5 Division of Epidemiology, College of Public Health, The Ohio State University, Columbus, Ohio, Unites States of America; 6 Epidemiology Division, Dalla Lana School of Public Health, University of Toronto, Toronto, Ontario, Canada; 7 Julius L. Chambers Biomedical/Biotechnology Research Institute, North Carolina Central University, Durham, North Carolina, United States of America; 8 Division of Public Health, Communicable Disease Branch, North Carolina Department of Health and Human Services, Raleigh, North Carolina, United States of America; 9 Department of Maternal and Child Health, Gillings School of Global Public Health, University of North Carolina at Chapel Hill, Chapel Hill, North Carolina, United States of America; University of California Irvine, UNITED STATES

## Abstract

In 2001, the primary and secondary syphilis incidence rate in rural Columbus County, North Carolina was the highest in the nation. To understand the development of syphilis outbreaks in rural areas, we developed and used the Bayesian Maximum Entropy Graphical User Interface (BMEGUI) to map syphilis incidence rates from 1999–2004 in seven adjacent counties in North Carolina. Using BMEGUI, incidence rate maps were constructed for two aggregation scales (ZIP code and census tract) with two approaches (Poisson and simple kriging). The BME maps revealed the outbreak was initially localized in Robeson County and possibly connected to more urban endemic cases in adjacent Cumberland County. The outbreak spread to rural Columbus County in a leapfrog pattern with the subsequent development of a visible low incidence spatial corridor linking Roberson County with the rural areas of Columbus County. Though the data are from the early 2000s, they remain pertinent, as the combination of spatial data with the extensive sexual network analyses, particularly in rural areas gives thorough insights which have not been replicated in the past two decades. These observations support an important role for the connection of micropolitan areas with neighboring rural areas in the spread of syphilis. Public health interventions focusing on urban and micropolitan areas may effectively limit syphilis indirectly in nearby rural areas.

## Introduction

Pronounced disparities for syphilis persist in the southeastern US, where discrimination against minorities, economic disadvantage, pervasive poverty, low quality education, limited access to health care, and residential and social segregation shape the social context [1–3]. Unlike other regions, rural areas of the Southeast have sustained high syphilis rates comparable to, or exceeding, rates in urban areas [4].

In 1999, the Centers for Disease Control and Prevention (CDC) launched the Syphilis Elimination program (SEP) to address the high burden of syphilis in a number of geographic areas including the southeastern US, targeting several states, including North Carolina [5]. In North Carolina, funding focused on the counties with the highest syphilis incidence rates (Mecklenburg, Wake, Durham, Guilford, Forsyth, and Robeson). Although the efforts of the elimination program initially showed success, in 2000, Robeson County, a largely rural county in southeastern North Carolina experienced a sharp rise in county-level rates of primary and secondary (P&S) syphilis, peaking at 71.7 cases per 100,000 person-years [6]. This incidence rate was more than 32 times higher than the national rate (2.2 cases per 100,000 of P&S in 2001), 21 times higher than the rate reported for the South (3.4/100,000 yearly person-time (p-y)), and 13 times higher than the overall rate reported for North Carolina (5.5/100,000) [6]. The next year, in 2001, Columbus County, a rural county with low population density bordering Robeson to the southeast, had the highest incidence of syphilis in the nation with 74.1 cases per 100,000 [2].

Similar to other syphilis outbreaks in the 1990’s, the syphilis outbreak in Columbus and Robeson counties was predominantly among heterosexual African-Americans [2] and was attributed, at least in part to crack cocaine use and exchange of sex for drugs [2, 7–9]. Sexual network analysis showed strong cohesion of the network structures, with dense interconnection and substantial cyclicity, or complete circular linkages among a set of sex partners [2, 8]. The sexual network analysis, however, did not reveal substantial connections between Robeson County and its rural neighbor to the south, Columbus County [8].

Hence, how the outbreak progressed to rural Columbus County is unclear, given the absence of defined sexual network connections between persons in Robeson and Columbus counties. This question is particularly critical because the Columbus outbreak occurred concurrently with the implementation of CDC’s Syphilis Elimination Program. Insight regarding the development and progression of this outbreak in a rural county in the midst of an active syphilis elimination program is useful to inform syphilis interventions today, given the persistence of syphilis epidemics in the rural south. Furthermore, previous network analyses showed limited connections across counties. When these connections were assessed temporally they were unlikely to have been the specific connection responsible for spread. This deficit likely reflects incomplete network analyses, given the inability even with strong contact tracing to determine every linkage in the communities.

Future technology that permits real-time visualization of network and outbreak progression on spatial maps, coupled with other details about cases, could expedite contact tracing and disease control. Our objective was to characterize the spatiotemporal development of the syphilis outbreak and observe the spread of infection through Robeson County and into Columbus County. We sought to determine whether the outbreak spread continuously across the two adjacent counties or occurred in distinct spatiotemporal clusters within each county. Although these data are now 20 years old, they remain highly relevant today, as this outbreak is one of the best characterized in terms of the underlying sexual networks. These data have served as the foundation for several of our studies as the contact tracing is in greater depth than most other data and thus the findings are highly valuable [9].

In addition, the critical questions of syphilis transmission mechanisms in rural areas remain. We hypothesized that the space/time mapping of incidence rates at the ZIP code and census tract level would provide information which, when combined with our previously reported sexual network analysis [8] would allow us to better understand the mechanism of propagation of the syphilis outbreak within a predominantly rural region. Our study addresses this hypothesis through the development of a series of Bayesian Maximum Entropy (BME) analyses of syphilis incidence rates.

While the mathematical framework of BME has been described [10–13], its implementation necessitates the use of a programming platform requiring specialized technical skills that may not be widely available at public health agencies. To address this issue we developed, and here demonstrate the use of a freely-available, shareware graphical user interface, which automates the BME map development and facilitates a disease mapping analysis. Disease incidence rates reflect uncertainties dependent on multiple factors including the data collection method (manual vs automated), number of observations available, population size, and level of aggregation. Additionally, incidence rates express high variability in space and time. This variability is especially relevant for Sexually Transmitted Disease (STI) outbreaks as outbreaks are often isolated to small geographic areas and specific time periods. Therefore the aggregation level and number of observations can have a large effect on the resulting incidence rates. In public health analyses, BME techniques can account for variabilities in rates resulting in more accurate results than other methods of geographical surveillance [14–17].

## Materials and methods

This study was reviewed by the University of North Carolina Committee on the Protection of the Rights of Human Subjects and was determined to be exempt, based on exemption criterion 4 (IRB 05–3080). Criterion 4 is an IRB exemption for secondary research, which may be done without consent if certain criteria are met. The data included all primary, secondary, and early latent stage syphilis cases reported to the North Carolina Division of Public Health between January 1, 1999 and December 31, 2004 for seven adjacent counties: Robeson, Columbus, Bladen, Brunswick, Cumberland, Hoke, and Scotland. Columbus is a rural county with a low population density (58.4 people/mile^2^, 54, 749 total in 2000 [18]). Robeson County (130 people/mile^2^, 123,339 total [18] is the closest county with a small urban town (Lumberton; 20,795 people in 2000) [18], considered micropolitan by the US census. Cumberland County (464.2 people/mile^2^, 302,963 total [18]) is the closest county categorized by the US Census with a metropolitan statistical area (Fayetteville, 121,015 people in 2000 [18]).

We back-estimated the date of syphilis exposure and infection (i.e. transmission date) for each syphilis case by subtracting the median latency period for the specific syphilis disease stage from the diagnosis date [19]. Primary syphilis cases were back dated 45 days, secondary syphilis cases were back dated 90 days, and early latent syphilis cases were back dated 183 days.

Case locations were geocoded with ArcGIS 9.3.1 [20] using self-reported residential addresses, and geomasked by the North Carolina Division of Public Health to preserve patient confidentiality. Satori Bulk Mailer software [21] was used to reformat and correct street addresses before geocoding to optimize the match rate of case addresses to current maps. Cases with a post office box address were spatially assigned to the post office address. Cleaned addresses were then matched to a location using address matching maps from: 1) the North Carolina Emergency Response System (E911), which contains point locations for all North Carolina households including rural routes; 2) the North Carolina Department of Transportation, which contains street-level geographic data; or 3) ESRI’s 2006 Street Map [22], which is primarily used for locating residences with outdated street names, prisons and military bases. Geomasking was performed using the donut method [16, 23]. The donut method relocates each geocoded data point within a random distance to ensure patient privacy and maintain spatial resolution necessary for cluster and outbreak detection.

Syphilis rates were estimated by spatially aggregating cases by ZIP code and census tract and temporally aggregating over rolling 6 month intervals to allow for the finest temporal aggregation with the sparsity of the dataset. Rates were then assigned to areal centroids. We denote *Y*_*ij*_ as the number of new syphilis cases observed in an area *i* with centroid location *s*_*i*_ over a duration *T* (0.5 years) centered at time *t*_*j*_. We calculate the 6-month areal incidence rate observed at location ***s***_*i*_ and time *t*_*j*_ as *R*_*ij*_ = *Y*_*ij*_/(*n*_*ij*_*T*) (cases per person-years), where *n*_*ij*_ is the population residing in area *i* at time *t*_*j*_.As all cases reported to the state were used in the analysis, space-time periods without syphilis records were given a rate of 0. ZIP code, census tract boundaries and population estimates were obtained from the US Census Bureau for the years 2000 and 2006. Populations for years between 1999 and 2004 were estimated by linearly interpolating/extrapolating the 2000 and 2006 census population estimates.

The mathematical framework of BME (see S1 Text) used in our mapping analysis has already been described in detail [11, 15, 16, 24]. In summary, BME uses principles of *maximum entropy* to process general knowledge about a Space/Time Random Field (S/TRF), and *Bayesian* epistemic data integration to blend site specific knowledge characterized by a variety of distributions including truncated log normal distributions [25–28], interval and normal distributions [16, 29], normal distributions [30, 31], log normal distributions [32], triangular distributions [33], or only hard data [34]. In the context of BME disease mapping (see S1 Text), BME methods are reduced to space/time simple kriging [2, 35] when observed incidence rates are treated as hard data (no uncertainties), and Poisson kriging when observed incidence rate are treated as Gaussian soft data (with uncertainties) such as the small number problem (population variability) [16].

The BME framework is implemented as follows: Let *F06D*(***s***,*t*) be a global mean trend function calculated for any spatial location ***s*** and time *t* in the mapping domain to capture global trends in disease rates. In short, the global mean trend is an exponential kernel smoothing function selected using an exploratory data analysis based on principles described in Nazelle et al. [36] to smooth local variability in the data and retain only global trends (see S1 Text for details). The global mean trend for this dataset has a spatial smoothing range set to 20 km and a temporal smoothing range set to 7 months.

Distinct covariance models were created for the ZIP code and census tract analyses. We define the transformation of incidence rates *z*_*d*_ observed at locations (***s***_*i*_,*t*_*j*_) (e.g. centroids of ZIP codes or census tracts) as *x*_*d*_ = *z*_*d*_–*F06D* (***s***_*i*_,*t*_*j*_). We then define *X*(***s***,*t*) as a homogenous/stationary space/time random field for which the transformed data *x*_*d*_ is a realization, and we let *Z*(***s***,*t*) = *X*(***s***,*t*)+ *F06D (****s***,*t*) represent the space/time random field of the incidence rate. We model the spatiotemporal autocorrelation of *X*(***s***,*t*) using the covariance function c_x_(*r*, *F074*), where *r* and *F074* are the spatial and temporal lags, respectively (See S1 Text for details on Syphilis Covariance models). The covariance function characterizes the autocorrelation in the transformed incidence rate data [37] *x*_*d*_ used by the simple and Poisson kriging methods to produce estimates of *X*(***s***,*t*) across the continuous mapping domain. By adding back the global mean trend *F06D* (***s***,*t*) to the estimated field *X*(***s***,*t*), we obtain maps of the incidence rate *Z*(***s***,*t*). Additional BME disease mapping approaches have been used in the past and are described in S1 Text.

Poisson kriging corrects the “small number problem”, whereby in administrative areas with small populations moderate changes in the number of cases lead to high variations in rates that visually dominate maps and may mask more relevant spatial patterns [38–40]. If the small number problem is an issue, Poisson kriging will smooth incidence rates observed on small populations (i.e. for small *n*_*ij*_ values). Conversely, if the small number problem is not an issue, Poisson kriging will not smooth observed incidence rates and the Poisson kriging maps will be the same as the simple kriging maps. Implementing both simple and Poisson kriging is useful to evaluate if the small number problem affects a specific mapping situation.

Syphilis rates were modeled and mapped at both the ZIP code and census tract levels, and using both simple and Poisson kriging, yielding four sets of spatiotemporal analyses. For each analysis, the steps included (1) an exploratory analysis of the data, (2) modeling the mean trend function to de-trend the data, (3) modeling the covariance function of the mean trend removed data, and finally (4) creating maps at different times to visualize the evolution of outbreaks. The outbreak duration was identified by qualitative review of epidemic curves for each county. While separate, repeated outbreaks are possible, the NC DHHS treated the event as a single interconnected outbreak. The health departments and disease intervention specialists working in the field recognized the outbreak was ongoing before the data could be processed.

To reduce the programming requirements of BME analyses, we implemented and tested the Bayesian Maximum Entropy geographical user interface software (BMEGUI version 2) (see S1 Text on BMEGUI implementation and https://mserre.sph.unc.edu/BMElab_web/index.htm). BMEGUI was designed by co-authors MS and YA, developed by YA, and tested in this study in the context of disease mapping, with all co-authors contributing to the critical review of disease estimates obtained. Results provided by a BMEGUI analysis are identical to BME analyses performed on other platforms such as MATLAB without necessitating knowledge of a programming language. BMEGUI’s interface is fully described in the user manual provided on the website for BMEGUI version 2 (https://mserre.sph.unc.edu/BMElab_web/index.htm). The syphilis rate estimations created by BMEGUI were then imported into ArcMap 9.3.1 [20] for a more aesthetic display of the estimated syphilis rates through space and time.

## Results

### Geocoding results

Of the 7,347 syphilis cases reported to the state during the outbreak between January 1, 1999 and December 31, 2004, 65% (4,773) were diagnosed as primary, secondary or early latent syphilis. These five years of data capture syphilis incidence rates across the state before, during and after the Robeson-Columbus outbreak. One fifth (21.6%, n = 1,032) of the cases originate from the seven counties included in this study. Although the state was experiencing an overall decline in syphilis incidence during the study period, the counties included in this analysis demonstrated rising incidence from June, 1999 through December, 2001 followed by a rapid decline. Approximately 75% (771) of the syphilis cases in the study area and time period were geocoded and matched to a location; 25% (261) did not match to a location. The most common reasons for failing to match to a location included: missing street data, military addresses, unhoused or out of state persons, rural routes with unavailable E911 addresses, and false addresses.

### Spatiotemporal mean trend and covariance models

**Fig 1:**
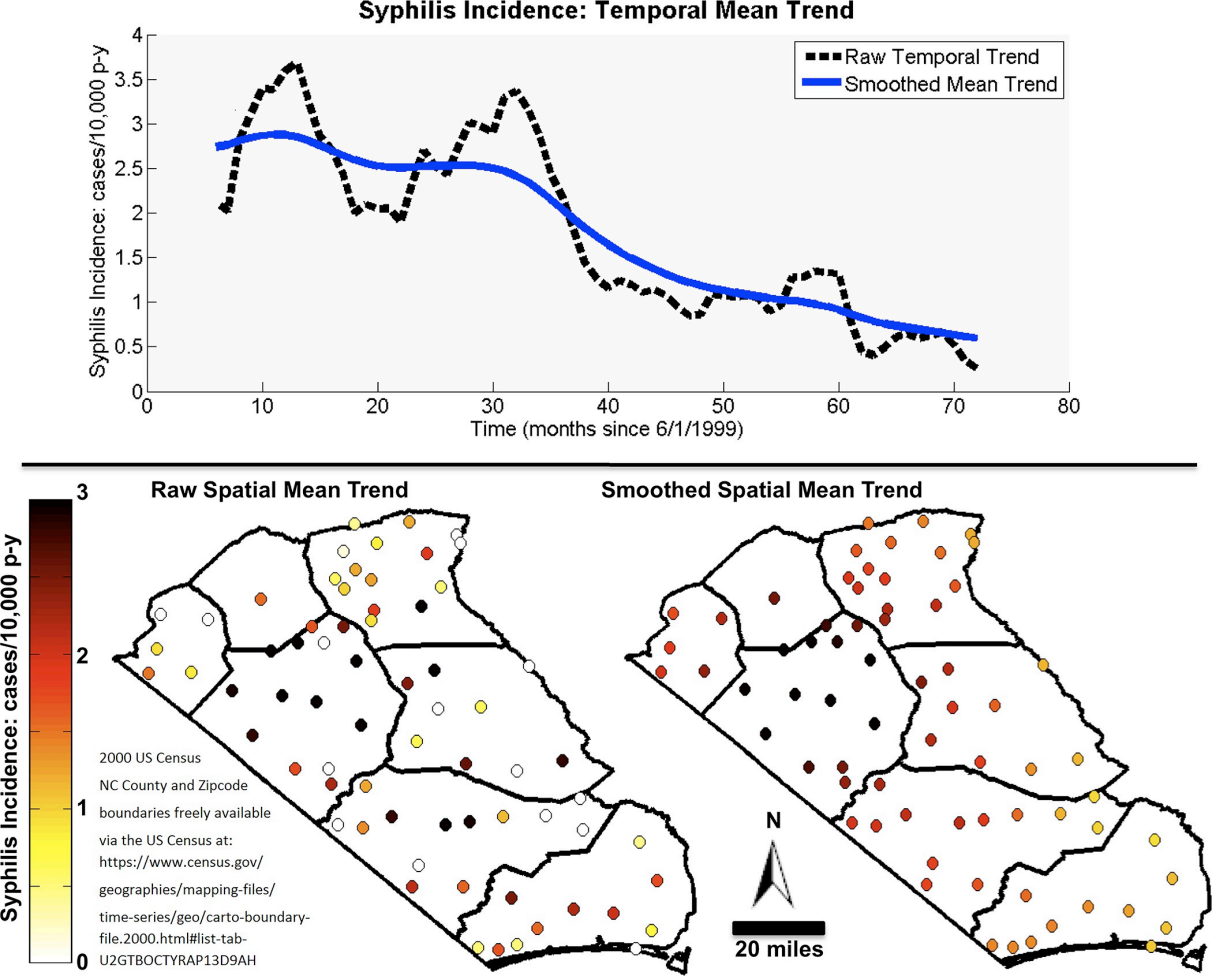
Spatial and temporal components of the mean trend of syphilis 6-month incidence rate (cases/person-years) over the Robeson Columbus outbreak area in 1999–2004. 2000 US Census North Carolina County, Census Track and Zipcode boundaries freely available via the US Census at: https://www.census.gov/geographies/mapping-files/time-series/geo/carto-boundary-file.2000.html#list-tab-U2GTBOCTYRAP13D9AH.

The ZIP code and census tract aggregations displayed comparable spatial and temporal mean trends (Fig 1). The covariance analyses show syphilis rates are most correlated for ZIP codes less than 10km apart and for census tracts less than 5km apart. This difference in distances is likely due to the differences between average ZIP code and census tract areas.

### Mapping results

**Fig 2:**
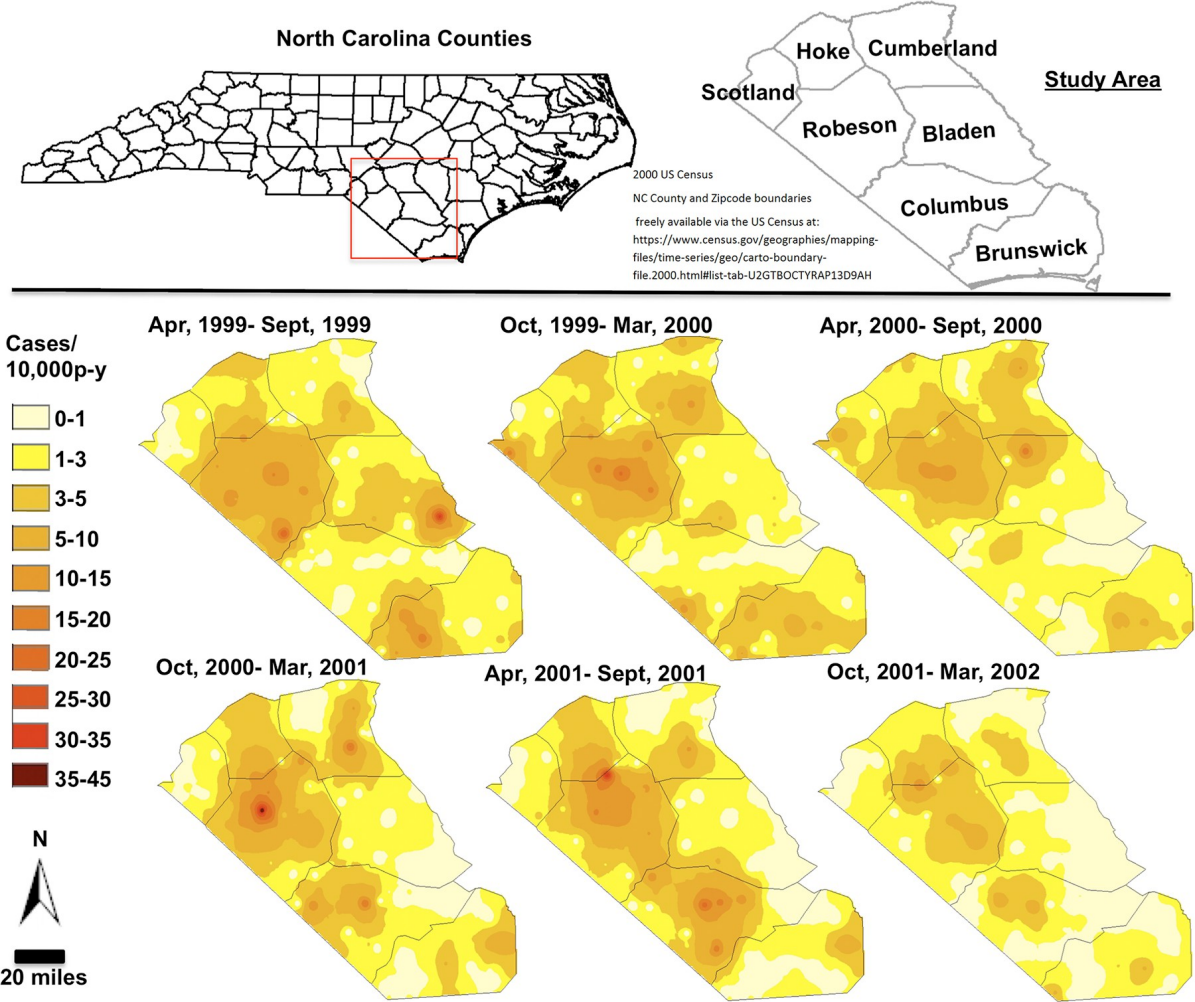
The location of the study area in North Carolina and the BME-simple kriging estimates of ZIP code-level syphilis incidence rates during the Robeson/Columbus outbreak from April 1999 to March 2002. 2000 US Census North Carolina County, Census Track and Zipcode boundaries freely available via the US Census at: https://www.census.gov/geographies/mapping-files/time-series/geo/carto-boundary-file.2000.html#list-tab-U2GTBOCTYRAP13D9AH.

As seen with simple kriging maps of ZIP code level syphilis incidence rates for six consecutive non-overlapping 6-month periods from April 1999 to March 2002, the outbreak began emerging in Robeson County in 1999 and persisted until 2002 (Fig 2). Cumberland County, to the northeast, had moderate syphilis rates throughout the period consistent with its endemic high case rate. Syphilis incidence rates were low in Columbus County initially (April 1999-September 1999 and October 1999-March 2000) followed by an outbreak in the third 6-month period (April 2000-September 2000). A spatial “corridor” of moderate rates (5–10 cases/10,000 person-years) connected Robeson and Columbus counties in the following two 6-month periods (October 2000-March 2001 and April 2001-September 2001), and dissipated in the last 6-month period (October 2001-March 2002). In the census tract analyses, areas with high rates had less connectivity than the zip code analysis, presumably due to the census tract’s finer geographic resolution. However, the similar spatial and temporal outbreak development patterns were seen in both the zip code and census aggregations and the Simple and Poisson kriging results (see S1–S3 Figs).

## Discussion

Publicly available surveillance data on syphilis are typically reported at a highly aggregated space/time scale (e.g. by county and year) to protect patient confidentiality. By taking advantage of geomasked data, our work refined the spatial resolution of publicly available maps to ZIP code and census tract and added a temporal component. The geostatistical analyses we conducted would typically require substantial programming expertise, particularly in the selection of the mean trend model, the covariance function, and the estimation methods. However, the BMEGUI tool greatly facilitated the implementation of the BME analysis without requiring programming skills. This tool allowed us to perform the BME analysis at two aggregation scales ensuring comparable conclusions.

We found the ZIP code and census tract aggregations had slightly different results but displayed similar patterns. As expected, for both aggregations the modeled global mean trend, *F06D*(***s***,*t*) = *F06D*_s_(***s***)+*μ*_t_(*t*) displayed smoother spatial and temporal variability than that of the observed local incidence rate data, *z*_*d*_, because the spatial smoothing range (20 km) and temporal smoothing range (7 months) were greater than the aggregation areas (ZIP code or census tract) and incidence time period (6 months). Consequently, the residual data *x*_*d*_ *= z*_*d*_*–F06D(****s***_*i*_,*t*_*j*_*)* for the residual S/TRF *X*(***s***,*t*) exhibited noticeable residual space/time variability modeled by the covariance function *c*_*x*_(*r*, *τ*). The temporal covariance showed cases are correlated over relatively long periods of time and reflects the persistence of disease transmission over time.

We found indistinguishable results using simple and Poisson kriging, supporting the conclusion that the small number problem was not an important issue for our study area. Generally, we recommend both methods be performed as results may vary between mapping situations. Although approximately 25% of the data could not be gecoded the missing data appeared to be missing at random without any noticeable patterns. We also believe the missing data had a minimal effect on the final results, adding only noise rather than a specific bias.

The data analysis took place much later than the outbreak occurred for several reasons. First, at the time of the outbreak the health department used an antiquated data management system created prior to relational databases. Furthermore, the technology (hardware and software) available at the time was also limited. Extensive efforts were expended to geocode the data in a secure data environment, securely geomask and finally aggregate the data in administrative boundaries requiring institutional review and approval of each step by the NC DHHS. The tracking software and data mining technology available today would have undoubtedly streamlined the process of creating an analytical dataset. Furthermore, the spatiotemporal methods were developed after the outbreak for targeted research questions applied here. The BMEGUI framework was also developed and adapted to disease mapping for this specific data set. As it was the first application of BME on these data, and given the sensitive nature of the data, extensive validation had to be conducted.

The recognition of the outbreak by the NC DHHS was accompanied by an extensive effort of contact tracing and social contact referrals with testing of those contacted. This effort, in combination with exhaustion of a susceptible population within the sexual networks, may have dissipated of the outbreak. The duration of the outbreak may have been lengthened by the density and size of the sexual network [8].

Our results are consistent with a study of a syphilis outbreak in Baltimore [10], which reported a leapfrog pattern of high incidence areas. In our context, extremely high and concentrated incidence areas in Robeson County were followed by high incidence areas in new, non-contiguous locations (e.g. Columbus County). Combining these results with our previous network analysis [8] provides a more comprehensive picture of the outbreak trajectory. First, our high resolution maps show the leapfrog jump of the outbreak from one county to its neighbor is followed by the establishment of a visible spatial corridor between these counties. Second, we learned from the socio-sexual network analysis that sexual network bridges linked individuals across some of these counties. Though our previous network analyses lacked temporal information, the combination of these findings provides a nuanced understanding of the mechanisms and transmission dynamics that propagated the outbreak: densely connected members of the sexual network geographically localized in Robeson County had sporadic bridges linking them with other densely connected persons in distinct geographical areas. In outbreaks driven by social networks where geographical proximity plays an important role, these bridges may predominantly link socio-sexual networks that are geographically close. These bridges may also lead to emergence of an outbreak in the newly connected sexual networks, leading to increasing rates in neighboring core areas and a visible corridor appears on the map.

This leapfrog-preceding-spatial-corridor mechanism explains how an outbreak can move to a rural area. In a previous syphilis cluster analysis in North Carolina we hypothesized the main pathway of STIs into rural areas may be through the interconnectedness of urban, micropolitan, small town, and rural areas [41]. Our findings support this hypothesis. Fayetteville, a major city in Cumberland County with a population of 121,015 in 2000 [21], had the highest counts of syphilis cases (and low rates) throughout the study suggesting it was a likely source of the outbreak. Infection moved from Cumberland to Robeson County where rates remained high throughout the study period (Fig 2). The epicenter of the Robeson outbreak appears to be an extension of the high endemic syphilis cases in Cumberland. Sexually connected persons in Robeson continued to transmit locally, and this group was geographically and sexually distinct from networks in other counties [8].

After the initiation of the outbreak in Robeson County, its micropolitan center, Lumberton, was critical in the outbreak expansion. Lumberton is the closest urban area easily accessible by rural residents of nearby Columbus County. This connection between a small town and proximal rural areas allows introduction of the outbreak into the rural sexual network. Consequently, the outbreak leapfrogged spatially into Columbus with subsequent development of geographical bridges as the outbreak expanded. Had the outbreak not been contained, these bridges may have coalesced into a larger inter-county outbreak, and then possibly a regional epidemic. Critically, this series of events implies that addressing STIs in urban and micropolitan areas will also indirectly help address STI rates in rural areas, as was suggested in our previous work [41].

We also believe the visible leapfrog jumps can act as an early warning sign an outbreak is about to expand, potentially spanning multiple counties. BME incidence rate maps can be used to identify these leapfrog jumps and subsequent spatial corridors to create more targeted and potentially cost-effective interventions to contain localized outbreaks before they expand into regional epidemics.

As with most studies using interpolation methods, our ability to visualize spatial corridors was impaired by a well-known kriging artifact referred to as “kriging islands”. Kriging is an exact interpolator; thus, at a data point the estimator has the same value as the data point. Kriging islands are seen in our maps as spots at aggregation (i.e. census tracts) centroids. At these kriging islands, the rates are either higher or lower than the surrounding areas. Furthermore, since ZIP codes and census tracts have arbitrary boundaries with greatly differing sizes, shapes, and populations, high incidence areas can either be displaced to the centroid or completely removed, if the cases occur on the border of two or more aggregation areas. Methods to overcome this limitation are needed. For example, geocoded cases aggregated on a regularly spaced, overlapping grid may provide better representations.

Our observation of the outbreak expanding through a geographical front coupled with temporal outward leapfrog jumps suggests novel techniques are needed to track fronts. An effective system to track fronts could act as an early alarm to inform public health officials that an outbreak is growing from a localized occurrence to a regional concern. Tracking fronts will require the development of methods to smooth disease rates and detect ramp ups preceding the emergence of an outbreak.

Although the data used for this analysis was twenty years old, the results remain relevant today. The combination of these spatial data with the previous extensive sexual network analyses, which are some of the most extensive syphilis network analyses, especially in rural areas, provides rich insights that have not been replicated in the past two decades. Our disease mapping approaches and analyses strengthen and expand findings from our previous sexual network analysis. However the previous analyses had slightly different data sources and substantial missing data. Furthermore, the previous sexual network analysis did not incorporate temporal information or fine scale geographical data. Finally, the methods used here provide useful examples that can be applied to other conditions, especially within rural areas. Future work should consider integrating space/time disease mapping with temporal analyses of sexual and social networks, while addressing the impact of missing data. Technology that permits real-time visualization of network and outbreak progression on spatial maps, coupled with other details about cases, could expedite contact tracing and disease control.

In conclusion, we observed a leapfrog pattern of syphilis transmission followed by development of local geographical bridges in a large outbreak in North Carolina. This outbreak with a large rural component appeared to be connected to both urban and micropolitan sources. When high disease incidence is seen in a relatively constrained geographic boundary and another geographically non-contiguous hot spot appears, it may suggest an outbreak is in progress as spatial linkages often do not appear immediately. Concentrated efforts to control an outbreak should be placed in densely populated areas as these resources will also substantially affect progression of an outbreak to less dense population centers. Future work must address the changing dynamics of syphilis, methods to monitor the leapfrog and bridging patterns of outbreaks, and the combination of social/sexual network and geospatial analyses.

## Supporting information

S1 Fig Spatiotemporal covariance *c*_*X*_(*r*,*t*) for the rolling 6-month syphilis incidence rate obtained using census track aggregated cases (left) and zip code aggregated cases (right). The spatial plots (top) show *c*_*X*_(*r*,*t* = 0) as a function of spatial lag *r*, and the temporal plots (bottom) show *c*_*X*_(*r* = 0,*t*) as a function of temporal lag *t*. Circles depict experimental covariance values, while the line depicts the covariance model.
(TIF)

S2 Fig Poisson kriging results for October, 2000- March, 2001 of the Zip code aggregated data set. (GIF)

S3 Fig Poisson kriging results for October, 2000- March, 2001 of the census tract aggregated data set. (GIF)

S1 Text (DOCX)
