# Supplementary figures and images for: Progression of a large syphilis outbreak in rural North Carolina through space and time: Application of a Bayesian Maximum Entropy graphical user interface
Source: PLOS Glob Public Health. 2023 May 4;3(5):e0001714. doi: 10.1371/journal.pgph.0001714 (PMC10159108; doi:10.1371/journal.pgph.0001714)

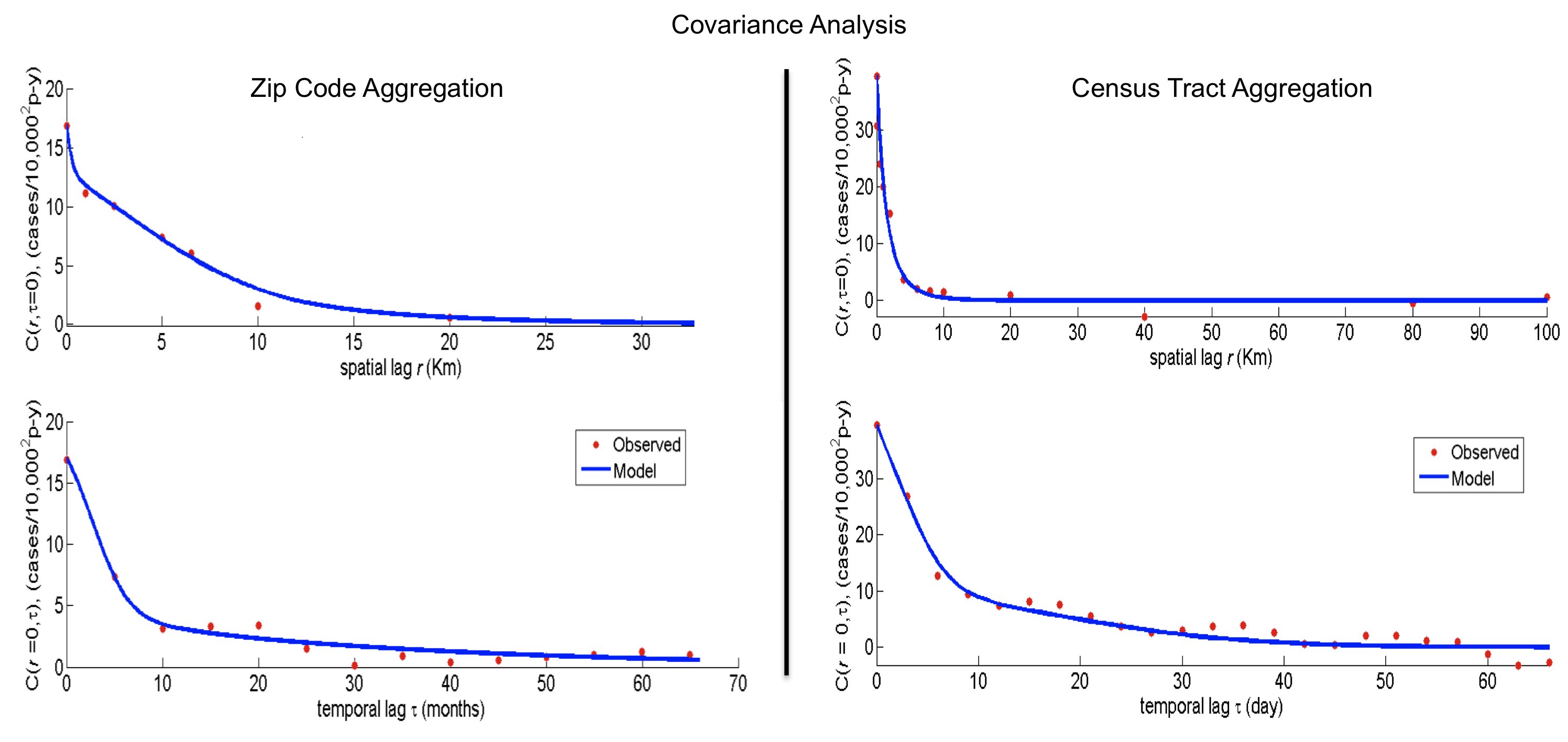

Supplement: S1 Fig — Spatiotemporal covariance cX(r,t) for the rolling 6-month syphilis incidence rate obtained using census track aggregated cases (left) and zip code aggregated cases (right). The spatial plots (top) show cX(r,t = 0) as a function of spatial lag r, and the temporal plots (bottom) show cX(r = 0,t) as a function of temporal lag t. Circles depict experimental covariance values, while the line depicts the covariance model. (TIF) [file pgph.0001714.s001.tif]

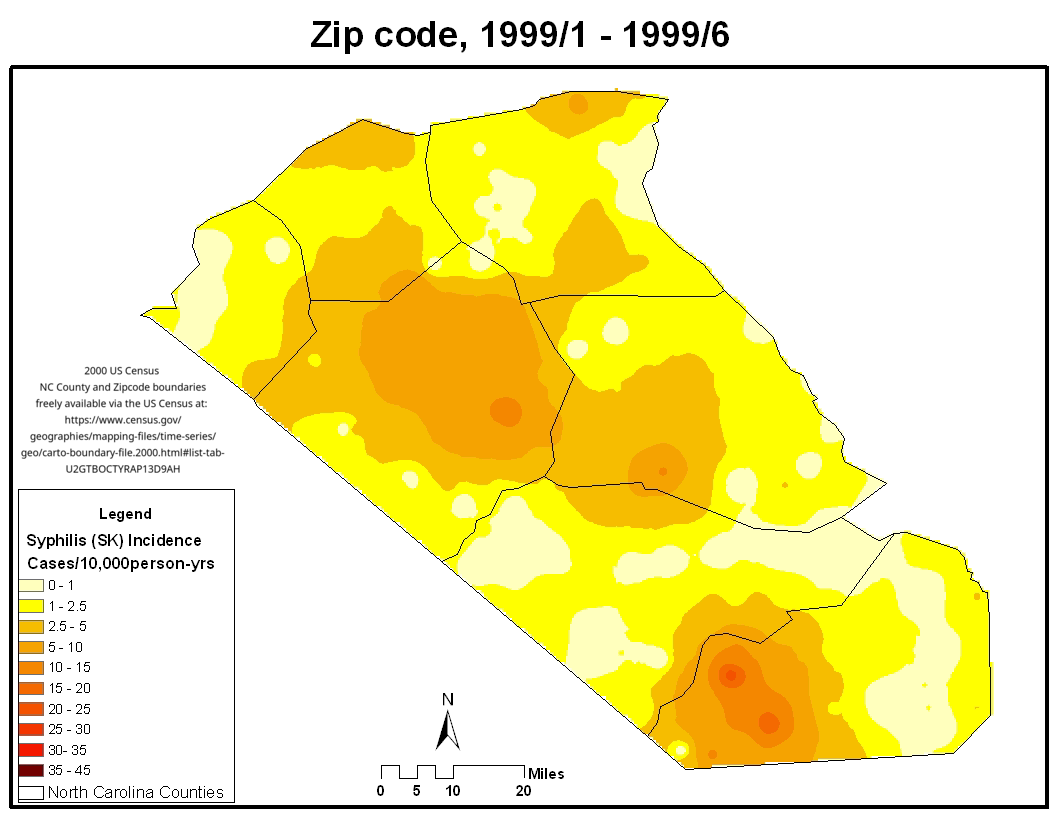

Supplement: S2 Fig — (GIF) [file pgph.0001714.s002.gif]

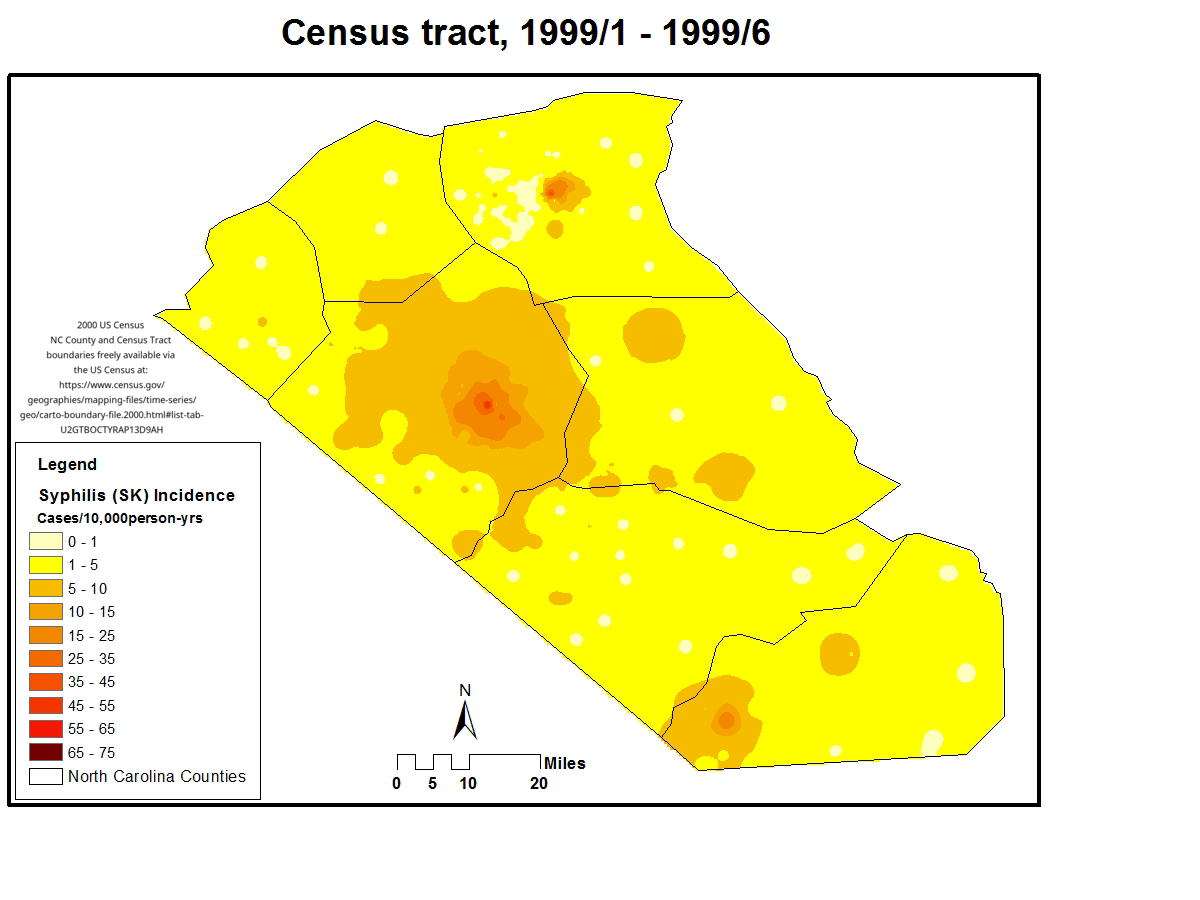

Supplement: S3 Fig — (GIF) [file pgph.0001714.s003.gif]
