# Supplementary material for: Progression of a large syphilis outbreak in rural North Carolina through space and time: Application of a Bayesian Maximum Entropy graphical user interface
Source: PLOS Glob Public Health. 2023 May 4;3(5):e0001714. doi: 10.1371/journal.pgph.0001714 (PMC10159108; doi:10.1371/journal.pgph.0001714)
Supplement: S1 Text — (DOCX) [file pgph.0001714.s004.docx]

Supporting Information

**Progression of a large syphilis outbreak in rural North Carolina through space and time: Application of a Bayesian Maximum Entropy graphical user interface**

Lani Fox, William C. Miller, Dionne Gesink, Irene Doherty, Kristen H. Hampton, Peter Leone, Del Williams, Yasuyuki Akita, Molly Fitch, Marc L. Serre

correspondence to: [marc_serre@unc.edu](mailto:marc_serre@unc.edu)

**This file includes:**

Supplementary Text

Figs. S1 to S3

Captions for Movies S1 to S2

**Other Supplementary Materials for this manuscript include the following:**

Movies S1 to S2

**Supplementary Text**

*The BME mathematical framework*

The Bayesian Maximum Entropy framework of modern spatiotemporal geostatistics has been presented in detail in previous works [12; 15; 16; 24]. The variability across space and time of a parameter *X* of interest is represented using the space/time random field (S/TRF) *X*(***p***) at space-time coordinate ***p***=(***s***,*t*), where ***s*** is the spatial coordinate and *t* is time. Capital letters (e.g. *X*) denote random values and lowercase (e.g. *x*) represent deterministic values, while boldface represent vectors of values, e.g. ***s***=[*s*_1_, *s*_2_], ***x***=[*x*_1_, …, *x*_n_], etc. In BME, the knowledge *K* about the S/TRF *X*(***p***) is organized in the general knowledge *G*={*m*_X_(***p***), *c*_X_(***p***,***p***’)} characterizing the mean *m*_x_(***p***)=E[*X*(***p***)] (E is the stochastic expectation operator) and covariance *c*_X_(***p***,***p***’)=E[(*X*(***p***)-*m*_x_(***p***)) (*X*(***p***’) -*m*_x_(***p***’))] of *X*(***p***) over the whole study domain, and the site specific knowledge *S*={***x***_h_, f_S_(***x***_s_)} consisting of data available at hard data points ***p****_h_* where *X* takes values ***x***_h_ known without error, and at soft data points ***p****_s_* where *X* takes values ***x***_s_ known with some uncertainty quantified by the site specific knowledge base pdf *f*_S_(***x***_s_). The three main steps of BME to estimate the value *x_k_* of *X* at estimation point ***p****_k_* consist of (1) using the information *Maximum Entropy* principles to process the general knowledge *G*, which results in a pdf *f*_G_(*x*_k_,***x***_h_,***x***_s_) that maximizes Shannon’s information entropy given the general knowledge, and (2) integrating the general knowledge base pdf *f_G_* with site specific knowledge *S* to obtain the pdf *f_K_*(*x*_k_), where the subscript *K* denotes the union of the knowledges *G* and *S*, using the *epistemic Bayesian conditionalization* rule expressed by the following equation

$$f_{K}\left( x_{k} \right)=A^{-1}\int{d\boldsymbol{x}}_{s}f_{G}\left( x_{k},\boldsymbol{x}_{h},\boldsymbol{x}_{s} \right)f_{S}\left( \boldsymbol{x}_{s} \right)$$

where $A=\int{dx}_{k}\int{d\boldsymbol{x}}_{s}f_{G}\left( x_{k},\boldsymbol{x}_{h},\boldsymbol{x}_{s} \right)f_{S}\left( \boldsymbol{x}_{s} \right)$ is a normalization constant, and (3) the interpretation stage where an estimate of *x_k_* and its associated estimation error are obtained by calculating the mean and variance of the BME pdf.

An attractive feature of BME is its highly successful ability to process complex space/time hard data with any type of soft data, including truncated log normal distributions [25-28], interval and normal distributions [16 29], normal distributions [30-31], log normal distributions [32], triangular distributions [33], or only hard data [34]. Another attractive feature of BME is that when only hard data are considered, then BME pdf reduces to [34]

$$f_{K}\left( x_{k} \right)=f_{G}\left( x_{k},\boldsymbol{x}_{h} \right)/f_{K}\left( \boldsymbol{x}_{h} \right)$$

with mean and variance equal to that of kriging. Similarly, if only normal distributions are used, then BME reduces to kriging with measurement error [16; 29-31].

*BME disease mapping methods*

Disease mapping consists in interpolating across space and time incidence disease rates observed at the centroid ***s****_i_* of areal administrative units and at time *t_j_*. The incidence rate observed at ***s****_i_* and *t_j_* is *R_ij_* = *Y*_ij_/(*n_ij_T*) (cases per person-years), where *Y*_ij_ is the number of positive disease cases observed amongst the population *n*_ij_ residing in area *i* at time *t_j_*. Three approaches have been described in previous BME disease mapping works [16; 29-31]: (a) disease rates can be assumed to be exactly equal to the observed rates, which results in a simple kriging interpolation of the observed disease rates *R_ij_*, (b) disease rates can be assumed to be in the interval [*R_ij_*-0.5/(*n_ij_***T*), *R_ij_*+0.5/(*n_ij_***T*)], which results in interpolating disease rates using a uniform distribution in these intervals and is referred to as the Uniform Model BME (UMBME) approach, and (c) case counts can be assumed to have a Poisson distribution [38], which results in interpolating disease rates using a Gaussian distribution with mean *R_ij_* and variance *m_j_**/(*n_ij_T*), where *m_j_** is the population-weighted mean of the observed rates for time *t_j_*, and is referred to as the Poisson kriging approach.

Space/time simple kriging is implemented by treating the observed incidence rates *R_ij_* as hard data assigned to area centroids ***s****_i_*, i.e. the data *z_d_* at locations (***s****_i_*,*t_j_*) is equal to *R_ij_* without error [10; 37]. Space/time Poisson kriging is implemented by incorporating observed incidence rates *R_ij_* treated as normally distributed “soft data” with an error variance equal to *m_j_**/(*n_ij_T*), where *m_j_** is the population-weighted mean of the observed rates for time *t_j_* [16;38].

*BMEGUI implementation*

Because of the versatility of the BME framework to integrate various types of soft data, in this work we have developed a BME Graphical User Interface (BMEGUI) that allows users to enter both interval as well as normal distributions for the soft data. Users can implement the simple kriging method by treating the observed rates as hard data. Users can implement the Poisson kriging method by using observed rates as Gaussian soft data, in which case BME reduces to kriging with measurement error. To convey that this method is obtained as a limiting case of BME using Gaussian data we refer to it as a BME kriging approach, though it could equally well be referred to as kriging with measurement error. Finally, users can implement the UMBME method by using soft data of uniform type. In that case BME does not reduce to kriging. The BMEGUI therefore offers a flexible platform to implement the simple kriging and Poisson kriging methods used in this work, as well as the UMBME method that is not used in this work but could be considered by some future users.

*Global Mean Trend*

We let *μ*(***s***,*t*) be a global mean trend function that can be calculated for any spatial location ***s*** and time *t* in the mapping domain. We define the transformation of the incidence rates *z_d_* observed at locations (***s****_i_*,*t_j_*) (e.g. centroids of zip codes or census tracts) as *x_d_*=*z_d_*–*μ*(***s****_i_*,*t_j_*). We then define *X*(***s***,*t*) as a homogenous/stationary Space/Time Random Field (S/TRF) for which the transformed data *x_d_* is a realization, and we let *Z*(***s***,*t*)=*X*(***s***,*t*)+*μ*(***s***,*t*) be the S/TRF representing the incidence rate. The global mean trend *μ*(***s***,*t*) at spatial coordinate ***s*** and time *t* was calculated as

*μ*(***s***,*t*)=*μ*_s_(***s***)+*μ*_t_(*t*)-, (1)

where *μ*_s_(***s***) is the spatial mean trend, *μ*_t_(*t*) is the temporal mean trend, and is the time average of *μ*_t_(*t*). BMEGUI calculates * μ*_s_(***s***) and *μ*_t_(*t*) using an exponential kernel smoothing of the time-averaged and spatially-averaged data, respectively, using kernel parameters that control how smooth the mean trend model is. Specifically, the spatial trend *μ*_s_(***s***) is calculated as , where *Z_i_* is the time-average disease rate at centroid *i*, is a weight that decreases exponentially as a function of the distance between the estimation point ***s*** and centroid ***s****_i_*, and *R* is the spatial smoothing range parameter. Similarly the time trend *μ*_t_(*t*) is calculated as , where *Z_j_* is the spatially-averaged disease rate for incidence period *j* and is an exponentially decreasing weight controlled by the temporal smoothing range parameter *T*.

BMEGUI automatically calculates a residual rate value *X_ij_* for each rate *Z_ij_* value observed at centroid ***s****_i_* and incidence period *t_j_* using the equation *X_ij_*=*Z_ij_*-*μ*(***s****_i_*,*t_j_*). The variance *v_X_* of the residual rate values captures the variability of the residual rate field *X*(***s***,*t*). The covariance of the residual rate field is defined as *cov*(*X*(***s***,*t*),*X*(***s****’*,*t’*))=*v_X_corr*(*X*(***s***,*t*),*X*(***s****’*,*t’*)), where *corr*(*X*(***s***,*t*),*X*(***s****’*,*t’*)) is the correlation between *X*(***s***,*t*) and *X*(***s****’*,*t’*). Hence the covariance captures both the variability and autocorrelation of the residual field. The covariance of the transformed residual data was estimated using

*cov*(*X*(***s***,*t*),*X*(***s****’*,*t’*))=*c_X_*(*r*,*τ*) (2)

where the covariance model *c_X_*(*r*,*τ*) is function of only the spatial lag *r*=||***s***-***s****’*|| and temporal lag *τ*=|*t*-*t’*| that separates the residual rates *X*(***s***,*t*) and *X*(***s****’*,*t’*). Plots of the covariance model were constructed as a function of spatial and temporal lags and can provide insight about the variability and autocorrelation in the residual disease rate.

*Syphilis Covariance models*

Distinct covariance models *c_x_*(*r*,*τ*) were created for the zip code and census tract analyses. The covariance model used in this work for the rolling 6-month syphilis incidence rate aggregated at the zip code level is given by

c_x_(r,𝜏)=c_01_exp(-3r/a_r1_)exp(-3𝜏/a_t1_)+c_02_exp(-3r/a_r2_)exp(-3𝜏^2^/a^2^_t2_)+c_03_exp(-3r/a_r3_)exp(-3𝜏^2^/a^2^_t3_)

where *c_01_=*4.2*10^8^ (cases/person-years)^2^, *a_r1_=*1 Km, *a_t1_=9* months, *c_02_=8.1**10^8^ (cases/person-years)^2^, *a_r2_=2*3 Km, *a_t2_=*13 months, *c_03_=4.7**108 (cases/person-years)^2^, *a_r3_=13* Km and *a_t3_=16* months (see Figure S1). The covariance model for the zip code analysis was constructed as the sum of three space/time separable components consisting of either Gaussian or exponential functions (Figure S1). The spatial plot of zip code covariance *c_X_*(*r*,*τ* =0) depicts a sharp decline in correlation for spatial lags ranging from 0 to about 10Km followed by a slow decline from about 10 Km until correlation approaches zero for a spatial lag of about 20 Km. The temporal plot of zip code covariance *c_X_*(*r*=0,*τ*) displays a sharp decline in correlation for temporal lags between 0 to 10 months followed by a slow decline from approximately 10 months until correlation approaches zero for temporal lags greater than about 65 months. The temporal covariance shows cases are correlated over relatively long periods of time and reflects the persistence of disease transmission over time.

The covariance model for the rolling 6-month syphilis incidence rate aggregated at the census tract level is given by

c_x_(r,𝜏)=c_01_exp(-3r/a_r1_)exp(-3𝜏^2^/a^2^_t1_)+c_02_exp(-3r/a_r2_)exp(-3𝜏/a_t2_)+c_03_exp(-3r/a_r3_)exp(-3𝜏^2^/a^2^_t3_)

where *c_01_=*9*10^8^ (cases/person-years)^2^, *a_r1_=*10 Km, *a_t1_=*44 months, *c_02_=1.6**10^7^(cases/person-years)^2^, *a_r2_=*3 Km, *a_t2_=*10 months, *c_03_=1.5**10^7^(cases/person-years)^2^, *a_r3_=*5 Km and *a_t3_=*9 months. The spatial plot (see Figure S1) of census tract covariance *c_X_*(*r*,*τ* =0) depicts a sharp decline in correlation for spatial lags ranging from 0 to about 3 to 5 km, followed by a slow decline from about 5 km until correlation approaches zero for a spatial lag of about 20 Km. Thus, syphilis rates are most correlated for census tracts with centroids less than 5 Km apart, mildly correlated for centroids that are 5-10 km apart, and minimally correlated for centroids >20 km apart. The temporal plot of census tract covariance (Figure S1) has similar time scale correlations to the zip code temporal covariance (Figure S1).

**Fig S1.**

Spatiotemporal covariance *c_X_*(*r*,*t*) for the rolling 6-month syphilis incidence rate obtained using census track aggregated cases (left) and zip code aggregated cases (right). The spatial plots (top) show *c_X_*(*r*,*t* =0) as a function of spatial lag *r*, and the temporal plots (bottom) show *c_X_*(*r*=0,*t*) as a function of temporal lag *t*. Circles depict experimental covariance values, while the line depicts the covariance model.

*Simple Kriging Movies of the Outbreak*

Our BMEGUI analysis built upon and confirmed the results of Doherty et al.’s (Doherty, 2012) Robeson/Columbus outbreak sexual network study. Both Doherty’s study and the BMEGUI results showed high intra-county and low inter-county connectivity. Thus it is unlikely from both study’s results the spatial corridors were facilitating inter-county syphilis transmission. The results of both studies show if outbreak conditions exist in neighboring counties the outbreaks can grow and coalesce between counties. The movies showing the progression of the outbreak demonstrates this phenomenon. An outbreak is located in Robeson County with smaller outbreaks in neighboring counties appearing, disappearing and connecting to the Robeson county outbreak. Initially in April-September, 2000 an isolated outbreak appears in Columbus County, and expands from October, 2000 to March, 2001 until the Robeson and Columbus outbreaks coalesce in April, 2001. This larger epidemic begins to subside in October, 2001. A spatial corridor is shown linking Robeson and Columbus counties. Although this spatial corridor exists it does not appear to facilitate the transmission from one county to another. Rather the spatial corridor appears after the transmission from Robeson to Columbus County has occurred.

Please visit <https://mserre.sph.unc.edu/BMElab_web/mappingStudies/Syp_RobColNC/index.htm> to watch two movies of the Robeson Columbus syphilis outbreak in 1999-2004. One movie demonstrates the results for the zip code aggregation and the other for the census tract aggregation. Both movies show the results of the simple Kriging analysis progressing quarterly using a rolling six-month incidence period. The results for simple Kriging and Poisson Kriging were similar thus movies were not created for the Poisson Kriging analysis. However, Figures S2 and S3 show the Poisson Kriging maps created for October, 2000 - March, 2001.

**Fig S2.**

Poisson Kriging results for October, 2000- March, 2001 of the Zip Code aggregated data set.

**Fig S3.**

Poisson Kriging results for October, 2000- March, 2001 of the Census Tract aggregated data set.

**Supplementary Material Movies**

The supplementary movies are available at the following website:

<https://mserre.sph.unc.edu/BMElab_web/mappingStudies/Syp_RobColNC/index.htm>

Movie S1

Simple Kriging results for the Zip Code aggregation. The movie progresses quarterly using a rolling six-month incidence period.

<https://mserre.sph.unc.edu/BMElab_web/mappingStudies/Syp_RobColNC/ZCodeSyp_SKriging_Jan1999-Dec2004.gif>

Movie S2

Simple Kriging results for the Census Tract aggregation. The movie progresses quarterly using a rolling six-month incidence period.

<https://mserre.sph.unc.edu/BMElab_web/mappingStudies/Syp_RobColNC/CTractSyp_SKriging_Jan1999-Dec2004.gif>
